# Exploring UK-wide public perceptions of the 'public good' use of data for research and statistics

**DOI:** 10.23889/ijpds.v9i5.2735

**Published:** 2024-09-18

**Authors:** Shayda Kashef, Mary Cowan

**Affiliations:** 1 Administrative Data Research UK (ADR UK); 2 Department for Science, Innovation and Technology

In the UK, access to sensitive public data for research or statistics must serve the public good. These uses of data underpin the creation of better evidence for decision-making, however, decisions about what public good means rarely involve the public.

We carried out a UK-wide public dialogue to better understand public perceptions of ‘public good’ use of sensitive data for research and statistics within a UK context. This included deliberative in-person and online workshops with 68 members of the general public.

24 hours of deliberative discussions were analysed and distilled into five main findings, which demonstrate that participants want the public to have a say in how the public good is defined in the context of decision-making on accessing sensitive data for research or statistics. The participants strongly expressed that the use of data for research or statistics should address inequalities in society and minimise harms; both of which could be partly achieved through meaningful public engagement. Participants also suggested that they would like to see more communication around the benefits of the use of data for research and statistics. Finally, they were broadly supportive of more data sharing under best practice safeguards, over and above existing legal frameworks.

Insights from deliberative discussions with members of the public on their perceptions of public good use and access of sensitive data is reciprocally beneficial. It demonstrates trustworthiness to the public by meaningfully involving them in decisions about their data and ensures organisations can maximise the public benefit of their work.

